# Supplementary material for: Hybrid Models and Biological Model Reduction with PyDSTool
Source: PLoS Comput Biol. 2012 Aug 9;8(8):e1002628. doi: 10.1371/journal.pcbi.1002628 (PMC3415397; doi:10.1371/journal.pcbi.1002628)
Supplement: Text S4 — Complete source code for the PyDSTool package (version 0.88.120504). Includes API documentation and help files linking to web pages. This file is identical to the current public release on Sourceforge.net. (ZIP) [file pcbi.1002628.s004.zip › PyDSTool/html/PyDSTool.Toolbox.PySCes_SBML-pysrc.html]

xml version="1.0" encoding="ascii"?


PyDSTool.Toolbox.PySCes\_SBML


| Home | Trees | Indices | Help | PyDSTool |
| --- | --- | --- | --- | --- |

Package PyDSTool ::
Package Toolbox ::
Module PySCes\_SBML

[hide private]

[frames] | no frames]

# Source Code for Module PyDSTool.Toolbox.PySCes\_SBML

```
  1  """ 
  2  PySCes interface code for systems biology modeling and SBML model markup. 
  3   
  4  This toolbox assumes you have PySCes installed. 
  5   
  6  R. Clewley, 2012 
  7  """ 
  8  from __future__ import division 
  9   
 10  from PyDSTool import Generator 
 11  from PyDSTool.common import args, remain 
 12  from PyDSTool.common import _seq_types, _num_types 
 13  import PyDSTool.Redirector as redirc 
 14   
 15  import numpy as np 
 16  from scipy import linspace, isfinite, sign, alltrue, sometrue 
 17   
 18  import copy, os, sys 
 19   
 20   
 21  # ---------------------------------------------------------------------------- 
 22   
 23  _functions = ['get_pysces_model', ] 
 24   
 25  _classes = [] 
 26   
 27  _features = [] 
 28   
 29  __all__ = _functions + _classes + _features 
 30   
 31  # ---------------------------------------------------------------------------- 
 32   
 33  try: 
 34      import pysces 
 35  except ImportError: 
 36      raise ImportError("PySCes is needed for this toolbox to work") 
 37   


38 -def make_varspecs(m, fnspecs):


39      # derived from PySCes.PyscesModel module, class PysMod.showODE method 
 40      maxmetlen = 0 
 41      for x in m.__species__: 
 42          if len(x) > maxmetlen: 
 43              maxmetlen = len(x) 
 44   
 45      maxreaclen = 0 
 46      for x in m.__reactions__: 
 47          if len(x) > maxreaclen: 
 48              maxreaclen = len(x) 
 49      odes = {} 
 50   
 51      for x in range(m.__nmatrix__.shape[0]): 
 52          odestr = '' 
 53          beginline = 0 
 54          for y in range(m.__nmatrix__.shape[1]): 
 55              reaction = m.__reactions__[y] 
 56              reaction_args = '(' + ','.join(fnspecs[reaction][0]) + ')' 
 57              if abs(m.__nmatrix__[x,y]) > 0.0: 
 58                  if m.__nmatrix__[x,y] > 0.0: 
 59                      if beginline == 0: 
 60                          odestr +=  repr(abs(m.__nmatrix__[x,y])) + '*' +  reaction + reaction_args 
 61                          beginline = 1 
 62                      else: 
 63                          odestr +=  ' + ' + repr(abs(m.__nmatrix__[x,y])) + '*' +  reaction + reaction_args 
 64                  else: 
 65                      if beginline == 0: 
 66                          odestr +=  ' -' + repr(abs(m.__nmatrix__[x,y])) + '*' +  reaction + reaction_args 
 67                      else: 
 68                          odestr +=  ' - ' + repr(abs(m.__nmatrix__[x,y])) + '*' +  reaction + reaction_args 
 69                      beginline = 1 
 70          odes[m.__species__[x]] = odestr 
 71   
 72      if m.__HAS_RATE_RULES__: 
 73          for rule in m.__rate_rules__: 
 74              odes[rule] = m.__rules__[rule]['formula'] #.replace('()','') 
 75   
 76      return odes

 77   


78 -def get_pysces_model(filename, target='Vode'):


79      path, fname = os.path.split(filename) 
 80      m = pysces.model(fname, dir=path) 
 81   
 82      max_t = np.Inf 
 83   
 84      parlist = m.__fixed_species__ + m.__parameters__ 
 85      pardict = dict([(pname, p['initial']) for pname, p in m.__pDict__.items()]) 
 86      varlist = m.__species__ # list ['s0', 's1', 's2'] 
 87   
 88      icdict = dict([(vname, v['initial']) for vname, v in m.__sDict__.items() if not v['fixed']]) 
 89      fixed_species = dict([(pname, p['initial']) for pname, p in m.__sDict__.items() if p['fixed']]) 
 90      pardict.update(fixed_species) 
 91   
 92      fnspecs = {} 
 93      for R in m.__reactions__: # list ['R1', 'R2', 'R3', 'R4'] 
 94          R_info = m.__nDict__[R] 
 95          #assert R_info['Modifiers'] == [] 
 96          assert R_info['Type'] == 'Rever' 
 97          arglist = [] 
 98          for reagent in R_info['Reagents']: 
 99              r = reagent.replace('self.','') 
100              if r in varlist: 
101                  arglist.append(r) 
102          arglist.sort() 
103          fnspecs[R] = (arglist, R_info['RateEq'].replace('self.','')) 
104   
105      varspecs = make_varspecs(m, fnspecs) 
106   
107      for fname, fspec in m.__userfuncs__.items(): 
108          # Don't know how these are implemented yet 
109          fnspec[fname] = fspec 
110   
111      dsargs = args(name=fname[:-3], 
112                    varspecs=varspecs, 
113                    fnspecs=fnspecs, 
114                    pars=pardict, 
115                    ics=icdict, 
116                    tdata=[0, max_t]) 
117   
118      genclassname = target + '_ODEsystem' 
119      try: 
120          genclass = getattr(Generator, genclassname) 
121      except AttributeError: 
122          raise TypeError("Invalid ODE solver type") 
123      return genclass(dsargs)

124   
125   
126  ################################################## 
127  #  CODE NOTES FOR FUTURE DEVELOPMENT 
128  ################################################## 
129   
130  #m.__events__ # list of ? 
131  # compartments will be ModelSpec objects 
132   
133  #m.__compartments__ # dict of ? 
134  #m.__eDict__ # dict of events 
135   
136  """ 
137  m.__nDict__ # dict 
138    = {'R1': {'Modifiers': [], 
139          'Params': ['self.k1', 'self.x0', 'self.k2'], 
140          'RateEq': 'self.k1*self.x0-self.k2*self.s0', 
141          'Reagents': {'self.s0': 1.0, 'self.x0': -1.0}, 
142          'Type': 'Rever', 
143          'compartment': None, 
144          'name': 'R1'}, 
145   'R2': {'Modifiers': [], 
146          'Params': ['self.k3', 'self.k4'], 
147          'RateEq': 'self.k3*self.s0-self.k4*self.s1', 
148          'Reagents': {'self.s0': -1.0, 'self.s1': 1.0}, 
149          'Type': 'Rever', 
150          'compartment': None, 
151          'name': 'R2'}, 
152   'R3': {'Modifiers': [], 
153          'Params': ['self.k5', 'self.k6'], 
154          'RateEq': 'self.k5*self.s1-self.k6*self.s2', 
155          'Reagents': {'self.s1': -1.0, 'self.s2': 1.0}, 
156          'Type': 'Rever', 
157          'compartment': None, 
158          'name': 'R3'}, 
159   'R4': {'Modifiers': [], 
160          'Params': ['self.k7', 'self.k8', 'self.x3'], 
161          'RateEq': 'self.k7*self.s2-self.k8*self.x3', 
162          'Reagents': {'self.s2': -1.0, 'self.x3': 1.0}, 
163          'Type': 'Rever', 
164          'compartment': None, 
165          'name': 'R4'}} 
166   
167  m.__pDict__ # dict of param values (not fixed species) 
168   = {'k1': {'initial': 10.0, 'name': 'k1'}, 
169   'k2': {'initial': 1.0, 'name': 'k2'}, 
170   'k3': {'initial': 5.0, 'name': 'k3'}, 
171   'k4': {'initial': 1.0, 'name': 'k4'}, 
172   'k5': {'initial': 3.0, 'name': 'k5'}, 
173   'k6': {'initial': 1.0, 'name': 'k6'}, 
174   'k7': {'initial': 2.0, 'name': 'k7'}, 
175   'k8': {'initial': 1.0, 'name': 'k8'}} 
176   
177  m.__sDict__ # dict of species descriptions (variable and fixed) 
178   = {'s0': {'compartment': None, 
179          'fixed': False, 
180          'initial': 1.0, 
181          'isamount': False, 
182          'name': 's0'}, 
183   's1': {'compartment': None, 
184          'fixed': False, 
185          'initial': 1.0, 
186          'isamount': False, 
187          'name': 's1'}, 
188   's2': {'compartment': None, 
189          'fixed': False, 
190          'initial': 1.0, 
191          'isamount': False, 
192          'name': 's2'}, 
193   'x0': {'compartment': None, 
194          'fixed': True, 
195          'initial': 10.0, 
196          'isamount': False, 
197          'name': 'x0'}, 
198   'x3': {'compartment': None, 
199          'fixed': True, 
200          'initial': 1.0, 
201          'isamount': False, 
202          'name': 'x3'}} 
203   
204  m.__uDict__ # dict of units 
205    =  {'area': {'exponent': 2, 'kind': 'metre', 'multiplier': 1.0, 'scale': 0}, 
206   'length': {'exponent': 1, 'kind': 'metre', 'multiplier': 1.0, 'scale': 0}, 
207   'substance': {'exponent': 1, 'kind': 'mole', 'multiplier': 1.0, 'scale': 0}, 
208   'time': {'exponent': 1, 'kind': 'second', 'multiplier': 1.0, 'scale': 0}, 
209   'volume': {'exponent': 1, 'kind': 'litre', 'multiplier': 1.0, 'scale': 0}} 
210      """ 
211  #m.__userfuncs__ # dict of ? 
212  #m.__functions__ # dict of ? 
213  #m.__piecewises__ # dict of ? 
214  #m.__rate_rules__ # list of ? 
215  #m.__rules__ # dict of ? 
216
```

  


| Home | Trees | Indices | Help | PyDSTool |
| --- | --- | --- | --- | --- |

|  |  |
| --- | --- |
| Generated by Epydoc 3.0.1 on Fri May 4 15:24:25 2012 | http://epydoc.sourceforge.net |
